# Transport and retention of silica nanoparticles in glass-bead columns: effects of particle size, type, and concentration of ionic species

**DOI:** 10.1038/s41598-023-51119-8

**Published:** 2024-01-06

**Authors:** Reza Daneshfar, Siavash Ashoori, Bahram Soltani Soulgani

**Affiliations:** Department of Petroleum Engineering, Ahvaz Faculty of Petroleum Engineering, Petroleum University of Technology (PUT), Ahvaz, Iran

**Keywords:** Crude oil, Nanoparticles, Hydrology

## Abstract

Silica nanoparticles (SiO_2_ NPs) have garnered substantial attention as versatile additives in saline fluids, finding application in areas like environmental remediation, wastewater treatment, enhanced oil recovery, and carbon geo-sequestration. Despite their potential, the intricate interaction between electrolyzed nanoparticles and porous media remains inadequately researched in these contexts. This study delves into the pivotal yet underexplored aspect of silica nanoparticle absorption behavior within porous media, a key determinant of their practical effectiveness. The research focuses on silica particles with dimensions of 10 nm and 50 nm, synthesized via hydrolysis and condensation of tetraethyl orthosilicate (TEOS) in methanol. Employing packed glass bead columns as a surrogate for porous media, the study unravels the complex mechanisms governing nanoparticle transport and deposition. Comprehensive investigations encompass variations in particle sizes, ionic strength, and ionic species, resulting in the examination of 48 distinct flooding scenarios. UV/Vis spectrophotometry is used to quantify nanoparticle concentrations in effluents, elucidating their transport behavior within the porous media. Concurrently, pressure drop alterations across the media serve as indicators of particle plugging and changes in permeability. Intriguingly, specific conditions involving a nanofluid comprising 50 nm silica nanoparticles and 10,000 ppm of magnesium chloride exhibit pronounced permeability reduction, offering potential insights for optimizing applications. Particularly noteworthy is the unique reduction in silica particle retention on glass bead surfaces as salinity increases, especially in the presence of magnesium sulfate. A concentration of 5000 ppm magnesium sulfate induces a log-jamming mechanism, resulting in an amplified final-to-intermediate permeability ratio. Experimental outcomes align with observations from scanning electron microscopy, improving understanding of porous media retention mechanisms. This study contributes to a deeper understanding of interactions between nanoparticles and porous media, paving the way for enhanced application strategies.

## Introduction

In recent years, numerous researchers have directed their focus towards the movement, interaction, and deposition of fluids, including suspensions, salt, and asphaltene, within porous media^1–3^. Simultaneously, nanoparticles have gained significant attention from various sources^4,5^. The displacement of nanoparticle suspensions within porous media is a pervasive phenomenon observed in both natural processes and engineering systems^6^. Illustrative examples encompass a diverse range of applications, such as the remediation of contaminated soil and groundwater^7^, CO_2_ sequestration in deep saline aquifers or depleted oil and gas fields^8^, tertiary oil recovery^9,10^, water retention in arid agricultural soils^11^, microfluidic logic control^12,13^, ink diffusion on paper^14^, and drug delivery within vascular networks^15^. Nanoparticles are transported into the reservoir or porous formations as nanofluids (also known as nano-formulations), wherein nanofluids represent a dispersion of nanoparticles within a base fluid, typically water^16–18^.

Nanoparticles show significant promise for various applications in the oilfield^19,20^. These include their utilization in sensing and imaging techniques^21^, enhancing mobility control^22,23^, improving drilling fluid properties^24,25^, treating produced fluids^26^, interacting with asphaltene^27–29^, addressing unconventional reservoir challenges^30^, and aiding in enhanced oil recovery (EOR) efforts^31–33^. The movement and attachment of nanoparticles in oilfields pose significant challenges due to complex local conditions, including high salinity, low permeability, and diverse rock properties^34^. When nanoparticles adhere to solid matrices like rocks, soil, or sediment, their concentration may diminish, leading to potential inefficiencies or ineffectiveness in practical treatments^35,36^.

The successful implementation of various applications demands nanoparticles to travel extended distances through reservoir rocks while experiencing minimal retention^37,38^. However, the extensive use of nanomaterials has led to a significant deposition of particles in the environment, posing a considerable risk of contamination^39^. Therefore, it becomes crucial to comprehend the mobility, sustainability, and ultimate destiny of these NPs to mitigate potential environmental and health hazards^40,41^. According to the work by Zhang and colleagues, it was suggested that silica nanoparticles exhibit both reversible and irreversible adsorption behaviors when in contact with solid surfaces^42^. The researchers conducted experiments involving nanofluids injected into columns filled with crushed sedimentary rock. According to Li et al.'s research, it was demonstrated that when silica nanoparticles were dispersed in NaCl electrolyte solutions, their retention in calcium carbonate (calcite) packed columns increased with higher solution ionic strength^43^. However, no retention of the nanoparticles occurred when they were injected in pure water or Na_2_SO_4_ electrolyte solutions. In a study conducted by Sameni and colleagues, a series of micromodel tests were carried out to explore the transport characteristics of various types of nanoparticles, including MgO, SiO_2_, and Al_2_O_3_. The researchers observed that introducing a nanofluid with a high concentration of nanoparticles resulted in a decrease in permeability, primarily attributed to the pore plugging process^44^. Kim et al. conducted research to examine the behavior of surface-treated silica nanoparticles when deposited in a sand pack column^45^. The findings indicated that when the calcium concentration exceeded 1% and the nanoparticle concentration was above 1%, the interaction led to the formation of highly viscous nanoparticle aggregates. These aggregates were then retained within the unconsolidated sand pack in situations where the hydrodynamic force was insufficient. Abhishek et al. demonstrated that when combining low-salinity water flooding with silica nanoparticle flooding, the adsorption of NPs onto sandstone surfaces resulted in notable benefits^46^. These advantages included a reduction in mineral dissolution, minimized ion exchange, decreased loss of cementing minerals, and improved flow resistance, all in comparison to using low salinity water (LSW) alone. Liu et al. conducted a comprehensive investigation into the transport and adsorption of silica nanoparticles in extended granular columns, with a particular focus on their interaction with carbonate substrates^47^. Their findings suggested that under alkaline and low-salinity conditions, the transport of SiO_2_ NPs in carbonate reservoirs was facilitated. Interestingly, the inclusion of salts in nanofluids had a positive effect on the adsorption of NPs. Specifically, bivalent CaCl_2_ exhibited greater adsorption compared to monovalent NaCl at an equivalent salt concentration of 0.01 M. In a study conducted by Arain and colleagues, the focus was on examining the reversible and irreversible adsorption phenomena of silica NPs on carbonate surfaces that were either oil-wet or water-wet, under conditions simulating a reservoir environment^48^. The outcomes indicated that the initial hydrophilicity of both the nanoparticles and the carbonate rock surface played a significant role in influencing the adsorption of nanoparticles onto the rock. Interestingly, when nanoparticles and rock surfaces had opposite charges, they exhibited an attractive force, leading to the formation of monolayers or even multilayers of nanoparticles on the rock surface.

The primary emphasis lies in the utilization of silica nanoparticles due to their numerous advantages for applications on a large scale in comparison to other nanomaterials^49,50^. These advantages include being eco-friendly, easy to prepare, non-toxic, and commercially available in a wide range of sizes^51–53^. Furthermore, their interfacial properties and surface functional groups, which range from hydrophobic to hydrophilic, can be tailored, providing a high degree of versatility in their application^10,54,55^. The current studies on nanoparticle migration in porous media are lacking in several key areas. These areas include the impact of ionic strength, particle size, and concentration, as well as validating NP transport mathematical models with reliable experimental data. The influence of ions, particularly the presence of divalent cations commonly found in oil reservoirs, on particle transportation remains unclear. While extensive research has been conducted on the effects of monovalent cations on NP transport behavior^56^, little attention has been given to the influence of divalent cations. It is hypothesized that divalent cations like Mg^2+^, Ca^2+^, and Ba^2+^ could be more effective in inducing nanoparticle destabilization and agglomeration, leading to a reduced transport rate compared to monovalent cations like Na^+^^57,58^.

Limited research has been conducted on the impact of particle size on NP transport^59^. Previous investigations with TiO_2_, Al_2_O_3_, and Fe^0^ NPs suggest that larger NPs tend to have higher retention rates^59–62^. However, drawing definitive conclusions is challenging due to agglomeration issues and varying ionic strength^63,64^. The role of particle size in NP transport remains uncertain. Some studies propose a critical particle size of 30 nm for inorganic particles, but research on stable particle sizes below 30 nm is scarce^56^. Similarly, there are conflicting reports on the effect of particle concentration on NP retention and transport. One study suggested that higher inlet concentrations lead to increased colloidal retention at ionic strength (IS) > 0.1 mM^65^. However, other research showed that higher input concentrations result in larger surface coverage for both low (1 mM) and high (100 mM) ionic strength, consistent with another study^56,66^.

The main objective of this study is to enhance our comprehension of NP movement in saturated porous media, with a focus on the mentioned concerns. To achieve this, diverse sizes of silica NPs were synthesized, and a series of experiments involving saturated column tests were performed. The investigation encompassed the following aspects: (1) analyzing how different concentrations of monovalent and divalent cations impact the instability and deposition of NPs in nanofluids on porous media surfaces; (2) assessing the influence of particle size on NP retention and transport in saturated porous media; and (3) comparison of the behavior seen in the flooding tests with observations and analysis of scanning electron microscope (SEM) images taken from the surface of the glass bead to better understand the mechanisms involved. Extensive research has been conducted on deep bed filtration, specifically focusing on the effects of various factors such as particle size, fluid velocity, and the physical properties of fine particles (which are in the micron size range) within porous media^67–69^. However, there is a notable gap in the research concerning the study of nanoparticle adsorption and transport behavior in pore formations. As a result, this paper unveils the findings of a study examining the retention and transport behavior of SiO_2_ nanofluids in porous media, taking into account different salinity conditions.

## Material and methods

### Preparation of porous media

The glass beads, sized between 22 and 37 mesh (450–800 µm) with an average size of 675 microns, underwent a thorough cleaning process involving acid wash, deionized water rinse, and oven-drying to eliminate metal oxides and impurities. Glass beads were widely used in studying fluid flow in porous media, chosen for their physical and chemical properties that closely resemble those of natural materials like sandstone, driven by the similarity to the silica-rich composition in geological formations^70,71^. The glass column's porous matrix was packed meticulously to maintain a constant permeability and porosity^62,72^. The dry glass beads were saturated with distilled water and added to the column in increments of 2 cm, with a layer of water on top. The vibration was applied using a vortex mixer for 4 min after each 2 cm layer to settle the beads while keeping the column vertical. The column length was 40 cm, aided by placing metal meshes on both ends. Permeability was calculated as 334 D using Darcy's law based on measured differential pressure. The determination of the glass beads porosity involved extracting the glass beads from the packed column and subsequently drying them in an oven. The strict packing procedure resulted in a constant pore volume of 2.00 mL and a corresponding porosity of 39.8% for different packings. Table 1 presents a comprehensive depiction of the constituents and their respective weight percentages comprising the glass bead compounds employed in this study.

**Table 1:** Composition and weight percentages of glass bead compounds.

Component	SiO_2_	Na_2_O	CaO	MgO	Al_2_O_3_
Weight percentage	90	4	3	2	1

### Determination of characteristics of synthesized nanoparticles

**Figure 1:**
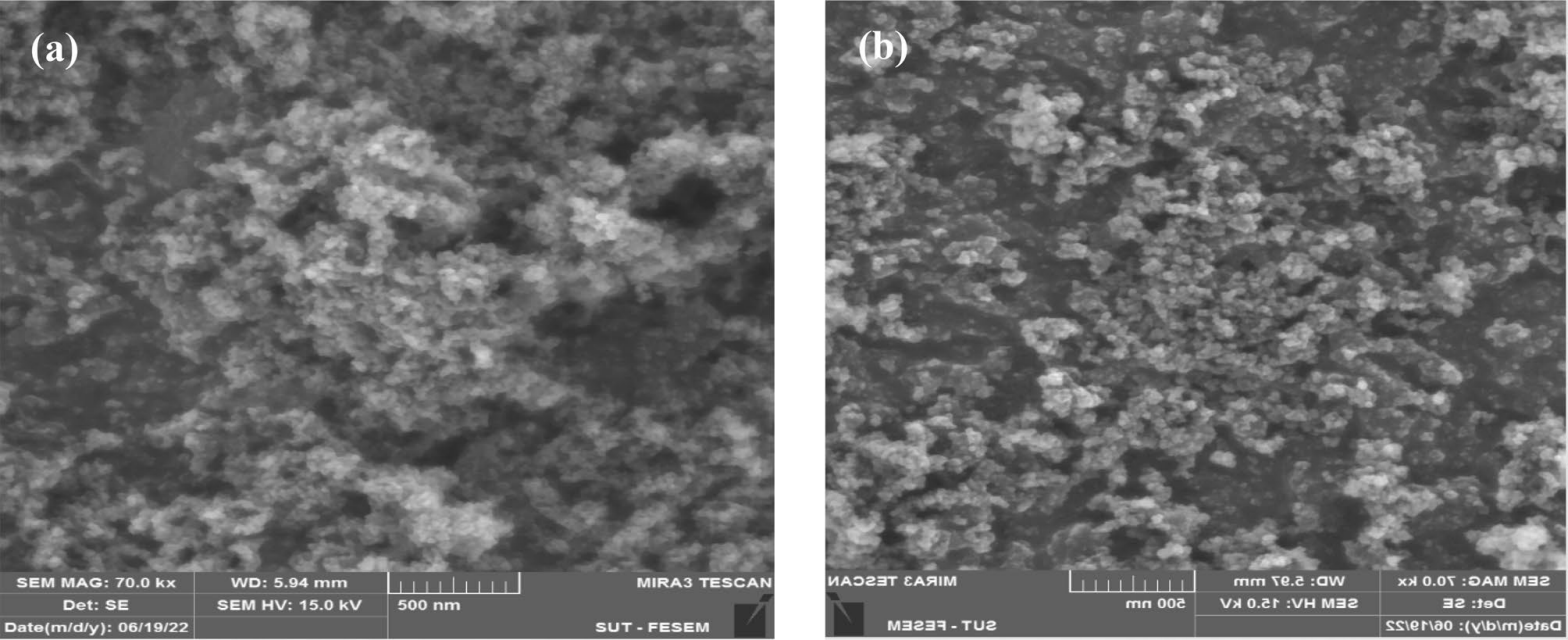
FESEM illustrations of synthesized silica particles, depicting dimensions of 10 nm in (**a**) and 50 nm in (**b**).

Silica particles of varying dimensions (10 nm and 50 nm) were produced via a hydrolysis technique, succeeded by tetraethyl orthosilicate condensation in methanol; further elaboration on this process can be found in another source^73^. In Fig. 1, the synthesized silica nanoparticles are meticulously characterized through high-resolution imaging using the field-emission scanning electron microscopy (FESEM) technique, specifically employing the MIRA3 Field Emission Gun scanning electron microscopy (FEG-SEM) by Tescan. The micrographs captured by this advanced imaging technology reveal the intricate morphology of the nanoparticles, highlighting their distinctive features. Part (a) of Fig. 1 exhibits silica nanoparticles with a diameter of 10 nm, as determined through comprehensive analysis using imageJ software. These meticulously characterized nanoparticles display a spherical morphology, indicative of their well-defined and uniform structure. In contrast, Part (b) showcases silica nanoparticles with a larger size, measuring 50 nm in diameter. The utilization of state-of-the-art FESEM, such as the MIRA3 FEG-SEM, not only emphasizes the precision in size determination but also provides valuable insights into the spherical nature of the synthesized nanoparticles, underscoring the efficiency of the synthesis process in producing particles of varying dimensions while maintaining a consistent and controlled morphology.

**Figure 2:**
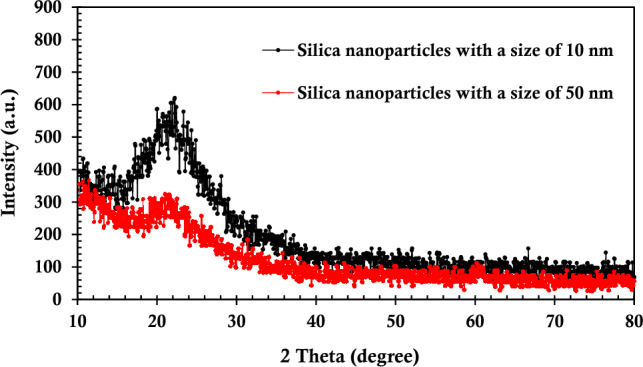
X-ray diffraction profile of the synthesized silica NPs.

In Fig. 2, the X-ray diffraction (XRD) analysis of the synthesized silica nanoparticles, characterized by distinct sizes, was conducted using the Philips PW1730 instrument with Cu-Kα radiation. The obtained XRD patterns offer valuable insights into the crystalline nature of the synthesized nanoparticles. Notably, a discernible difference emerges in the peak profiles, particularly evident in the sharper peak associated with the smaller nanoparticles. This heightened sharpness suggests an enhanced level of crystallinity in the smaller particles, underscoring the precision of the synthesis process^74^. The 2θ values derived from the XRD spectra contribute to the understanding of the crystallographic characteristics of the nanoparticles. The XRD scans were performed at a controlled scan rate, providing a comprehensive examination of the crystalline structure. The results highlight the effectiveness of the synthesis method in producing silica nanoparticles with superior crystalline properties, indicative of their high-purity nature.

**Figure 3:**
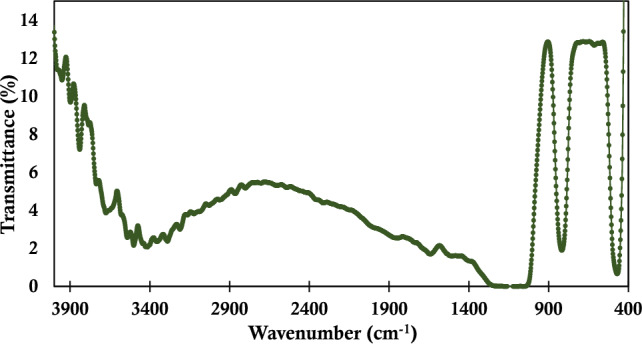
Raman spectroscopy results for the synthesized silica particles.

In Fig. 3, the Fourier-transform infrared spectroscopy (FTIR) analysis of the synthesized silica NPs was carried out to unravel insights into their molecular composition. The investigation was conducted using the BRUKER Tensor 27 instrument from Germany, known for its high-resolution capabilities. The FTIR spectra, focusing on a specific region with meticulous resolution, revealed key peaks associated with the structural characteristics of the synthesized nanoparticles. Notably, distinct peaks at 462 cm^−1^ and 801 cm^−1^ were identified and are attributed to the Si–O–Si vibrations, emblematic of the silica matrix^75^. These peaks confirm the successful synthesis of the nanoparticles and provide a clear indication of their integration into the silica network. Additionally, the presence of peaks in the hydroxyl region further corroborates the molecular structure of the synthesized nanoparticles. The careful examination of these FTIR spectra not only validates the synthesis process but also sheds light on the specific molecular bonds, particularly the Si–O–Si vibrations and hydroxyl functionalities, contributing to a comprehensive understanding of the synthesized silica nanoparticles.

### Preparation of nanofluid samples

This section outlines the process of preparing nanofluid samples for conducting experiments. Initially, brine solutions were created with varying concentrations (500, 1000, 2500, 5000, 7500, and 10,000 ppm) of NaCl, MgSO_4_, MgCl_2_ (supplied by Merck), and CaCl_2_ (supplied by Samchun). The salts were dissolved in deionized water using a magnetic stirrer for an hour at 500 rpm. The selected salt concentrations were chosen to study nanoparticle interactions, behavior, stability, and deposition characteristics under different salinity levels. Various nanofluids were then prepared by combining distinct silica nanoparticles of sizes 10 and 50 nm with brines to achieve a nanofluid concentration of 0.2 wt%. The mixtures were sonicated for 30 min using a 300-W Hielscher ultrasonic homogenizer (model UIP500 hd) to achieve uniform suspension. The Mettler mass balance was used to precisely determine the masses of both the salt and silica nanoparticles, achieving an accuracy level of 10^−5^ g. The 0.2 wt% nanoparticle concentration was chosen based on its common usage in previous studies for flooding, zeta potential, and particle size analyses^76–78^. In total, 48 nanofluids were prepared by incorporating these nanoparticles, enabling their utilization in various experimental procedures involving six concentration levels for each of the four types of salt. At a constant temperature of 25 °C, the fluids' viscosity was determined using distinct instruments. The DV3T-Brockfield viscometer determined viscosity measurements under normal room temperature conditions.

### Adsorption experiments in a glass bead column

**Figure 4:**
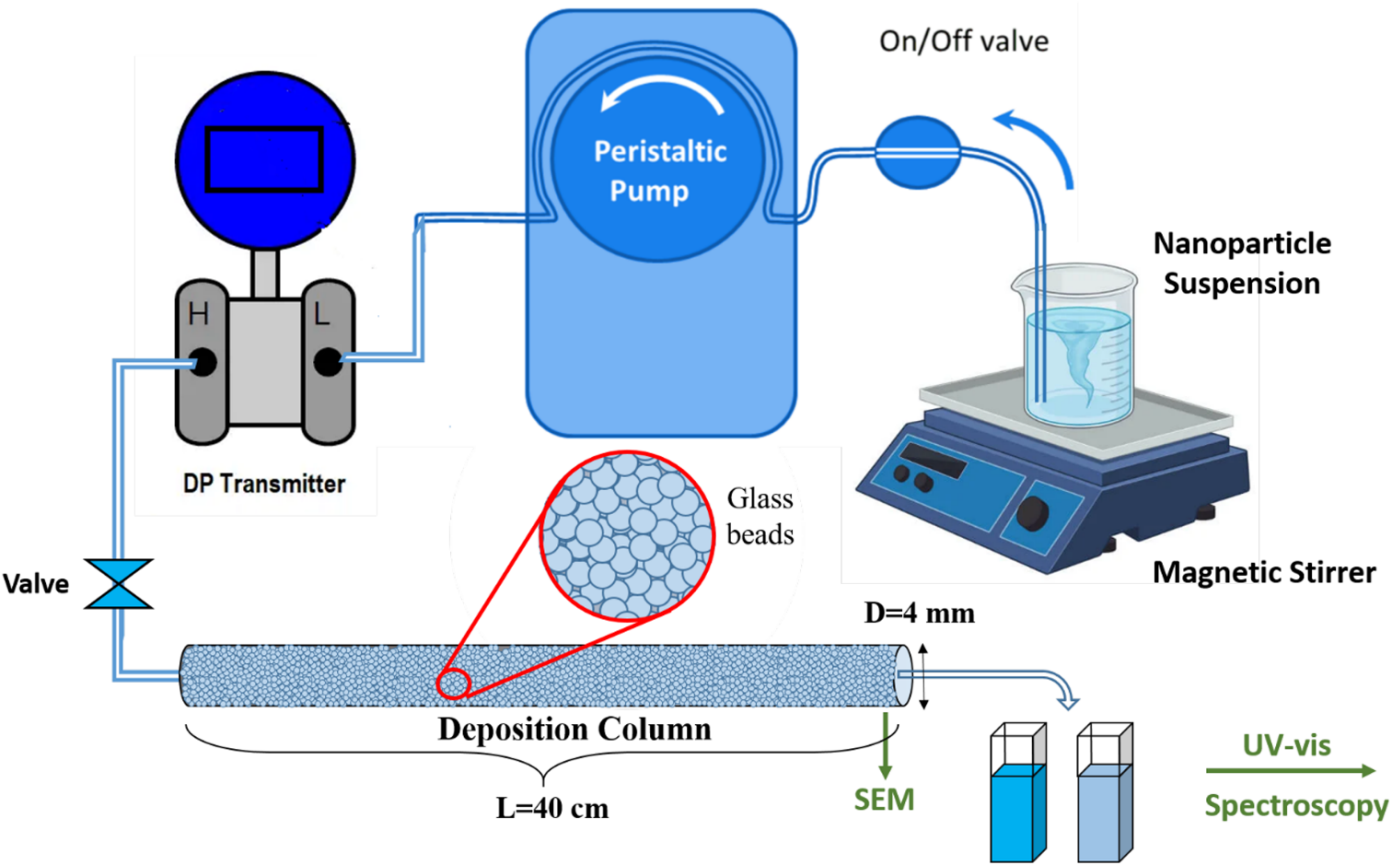
Schematic depicting the components of a deposition column.

To investigate transport characteristics in a deposition column (depicted in Fig. 4), experiments were conducted using dispersions of silica nanoparticles flowing through a transparent glass cylindrical column filled with glass beads. The adsorption column was intentionally maintained in a horizontal position for all 48 experimental scenarios to reproduce conditions commonly encountered in subsurface applications. This horizontal orientation aimed to isolate and study the intricate interplay between particle size, salt type, and concentration without the confounding effects of gravitational settling, which could become a significant factor when dealing with the transport and deposition of SiO_2_ aggregates. In all tests, the porous media were purged of air using a vacuum pump before injecting the fluid to prevent the entry of air bubbles. The experimental setup included a peristaltic pump (Hei-FLOW Gold model) operating at a constant flow rate of 0.326 mL/min, along with a pulse damper to reduce pulsations. The reason for choosing this low constant flow rate for the experiments was to ensure laminar flow conditions, closely resembling the characteristics of underground water systems^79,80^. To regulate viscosity, all tests were conducted at a consistent temperature of 25 °C, and permeability was determined using Darcy's law. The column apparatus comprised an adsorption glass column (40 cm in length and 4 mm in inner diameter), a UV-spectrophotometer (UV 1800, Shimadzu, Kyoto, Japan) for measuring nanoparticle concentration in the effluent collector, and an electronic differential pressure transmitter (150 psi, Yokogawa EJA110, with 0.065% accuracy).

During each experiment, the nanoparticles in a container with a magnetic stirrer were stirred at 500 rpm to maintain a constant concentration of nanofluid as it entered the porous media. The calibration tests demonstrated a linear relationship between nanoparticle concentration and absorbance at a wavelength of 800 nm. Transport and retention properties of silica nanoparticles were evaluated through 48 experiments conducted in pre-saturated glass columns. These properties were studied under the influence of particle size, salt type, and ionic concentration.

All the column experiments adhered to the same protocol, involving the following steps: (1) the glass column was dried, vacuumed, and saturated with brine having the same salinity as the nanoparticle dispersion. (2) Next, 5 pore volumes of the same saline water are injected into the porous media at a constant flow rate. (3) Then, a total of 500 pore volumes of the nanoparticle dispersion were injected into the deposition column. (3) After each transport test, a portion of the glass beads from the end of the column was removed, dried at 100 °C overnight, and preserved for SEM testing using a Quanta 600 FEG microscope. (4) Subsequently, the remaining glass beads were completely removed from the column, and new glass beads were packed into the column for the next experiment.

The Quanta 600 Field Emission Gun microscope, a cutting-edge scanning electron microscope developed by Thermo Fisher Scientific, was renowned for its exceptional imaging capabilities. Operating on the principle of field emission, it produced a focused electron beam with sub-nanometer resolution, enabling detailed visualization of nanoscale structures.

For every flooding scenario, subsequent to the injection of 5 pore volumes (calculated as the product of the porous media's total volume and porosity) of saline solution, a consistent flow of 500 pore volumes of suspension is introduced into the porous media. The normalized permeability, defined as the ratio of the permeability obtained in the third step to that obtained in the second step, is determined after completing each injection scenario. The purpose of normalizing permeability (dividing permeability by the initial permeability) is to mitigate errors arising from variations in pore size distribution during the preparation of identical porous media. This normalization facilitates a more accurate comparison of the performance across different scenarios, a practice widely adopted by previous researchers^81–83^.

## Results and discussion

### Analysis of normalized permeability values of the porous media after injection of suspensions

**Figure 5:**
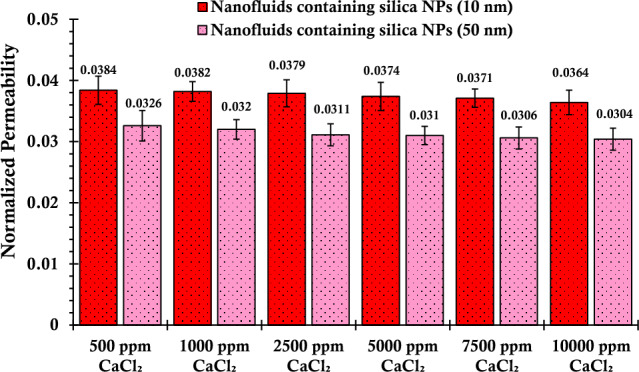
Normalized permeability values of porous media after injecting suspensions containing 0.2 wt% of SiO_2_ nanoparticles (with two different nanoparticle sizes) and varying concentrations of CaCl_2_.

This section explores the outcomes related to the injection of silica nanofluids, which have been prepared to use varying sizes of silica nanoparticles while also being subjected to diverse concentrations of salts. The injection of silica nanofluid in the presence of varying concentrations of calcium chloride salt in a glass bead column revealed a marginal increase in silica nanoparticle adsorption on the glass bead surface, which subsequently led to a slight decrease in the normalized permeability of the porous media, as depicted in Fig. 5. Evidently, an escalation in the salinity of calcium chloride salt within suspensions incorporating silica nanoparticles, ranging from 500 to 10,000 ppm, results in a noticeable decrease in permeability. For smaller-sized nanoparticles, the normalized permeability experiences a decline from 0.0384 to 0.0364, while larger-sized nanoparticles display a decrease from 0.0326 to 0.0304. The observed behavior can be elucidated through several mechanisms^84^: firstly, van der Waals forces between the silica nanoparticles and the glass bead surface played a role in facilitating the adsorption of nanoparticles onto the solid surface. Secondly, enhanced adsorption could be facilitated by electrostatic interactions between the negatively charged silica nanoparticles and the positively charged calcium ions present in the calcium chloride salt. Thirdly, hydrodynamics factors, such as fluid flow velocity and pore geometry, influenced the transport and distribution of nanoparticles within the porous media, influencing the extent of nanoparticle adsorption. Additionally, the behavior of nanoparticles inside the pores could be affected by the straining or log-jamming mechanism^85^, where nanoparticles experience hindered mobility or became trapped within narrow pore throats, reducing their overall transport and permeability of the porous media^86^. Gravitational settling is another crucial mechanism influencing this process. Despite the horizontal flow of nanofluids within the porous medium, once nanoparticles experience instability and aggregation, sedimentation may occur if the aggregate size surpasses the threshold where gravitational forces become significant^87^.

**Figure 6:**
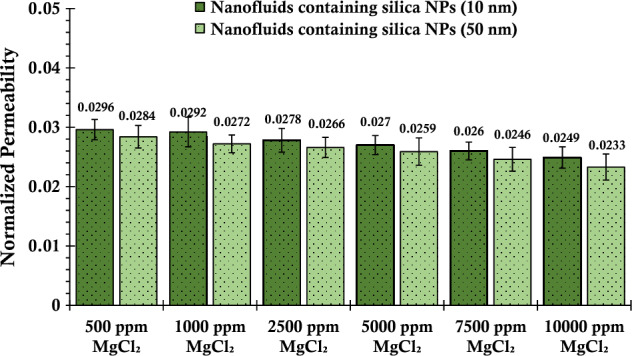
Normalized permeability values of porous media after injecting suspensions containing 0.2 wt% of SiO_2_ nanoparticles (with two different nanoparticle sizes) and varying concentrations of MgCl_2_.

In the following, experiments were conducted to investigate the effect of different concentrations of magnesium chloride salt on the injection of silica nanofluid into a column of glass beads, as shown in Fig. 6. The results revealed a notable increase in the absorption of silica nanoparticles onto the surface of the glass beads with the rise in salt concentration, consequently leading to a drastic reduction in the permeability of the porous media. The observed phenomenon can be attributed to the inherent instability of silica nanoparticles in the presence of magnesium ions, which arises from the interplay of van der Waals attraction and electrostatic repulsion forces. The interaction between silica nanoparticles and the porous media's surfaces, termed nanoparticle-pore interaction, facilitated the surface adsorption of silica nanoparticles on the glass beads. This adsorption process was further influenced by hydrodynamic forces and multi-particle plugging, ultimately contributing to the observed decrease in permeability^88^.

**Figure 7:**
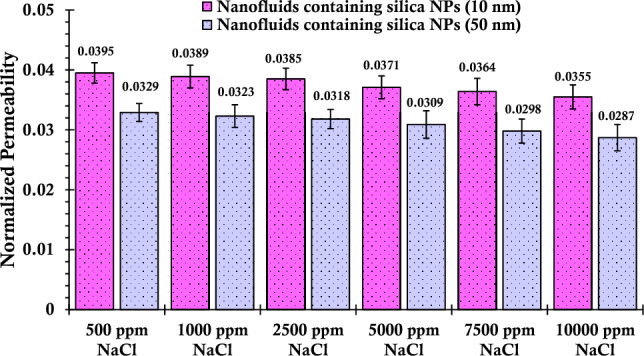
Normalized permeability values of porous media after injecting suspensions containing 0.2 wt% of SiO_2_ nanoparticles (with two different nanoparticle sizes) and varying concentrations of NaCl.

By comparing the outcomes of normalized permeability assessment for the porous media post the introduction of suspensions containing sodium chloride salt (Fig. 7) with the corresponding results derived from nanofluids infused with calcium chloride salt (Fig. 5), a notable observation comes to light. The inclusion of sodium ions appears to impart a heightened normalized permeability to the glass bead-laden column, particularly evident in situations of lower salinities. However, as the concentration of sodium chloride salt in the nanofluid escalated toward 10,000 ppm, a distinct trend unfolds. In this scenario, the formed aggregates tend to amass within the porous media, resulting in a reduction in permeability. This decline culminates in a normalized permeability measurement of 0.0287 for the formulated nanofluid. Notably, for nanofluids featuring smaller-sized nanoparticles, the normalized permeability ultimately attains a value of 0.0355.

**Figure 8:**
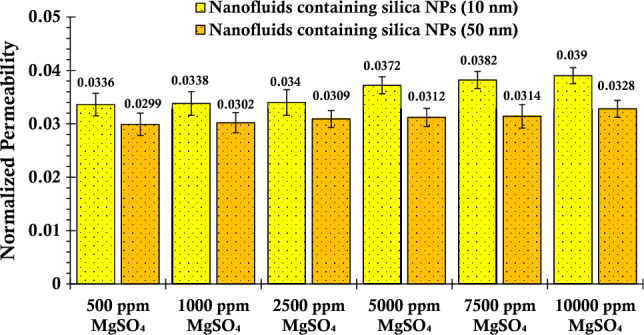
Normalized permeability values of porous media after injecting suspensions containing 0.2 wt% of SiO_2_ nanoparticles (with two different nanoparticle sizes) and varying concentrations of MgSO_4_.

In the following, experiments were conducted to investigate the effects of various concentrations of magnesium sulfate salt on the injection of silica nanofluids into a glass bead column, as shown in Fig. 8. The results shown in this figure revealed a notable decline in the adsorption of silica nanoparticles onto the glass bead surface with increasing salt concentration. Consequently, the permeability of the porous media exhibited a relatively lesser decrease. The observed behavior can be attributed to two underlying mechanisms. Firstly, the presence of magnesium sulfate salt introduced competition between salt ions and silica nanoparticles for available adsorption sites on the glass bead surface, leading to reduced silica nanoparticle deposition. Secondly, due to the opposite surface charges, electrostatic repulsion between silica nanoparticles and the glass bead surface hindered their retention. These mechanisms collectively contributed to the diminished impact on the porous media's permeability, providing valuable insights into nanoparticle transport behavior in porous media and implications for applications in enhanced oil recovery and groundwater remediation.

### Analysis of effluent concentrations of injected nanofluids

In this segment, we present and analyze the outcomes pertaining to the concentration of the effluent fluid extracted from the glass bead column during the final pore volumes of the suspensions introduced into the porous media. It is imperative to highlight that the concentration values reported herein were derived using the calibration curve, meticulously constructed via the UV spectroscopy technique. Comprehensive insight into the measurement details of this method can be found in "Preparation of nanofluid samples".

**Figure 9:**
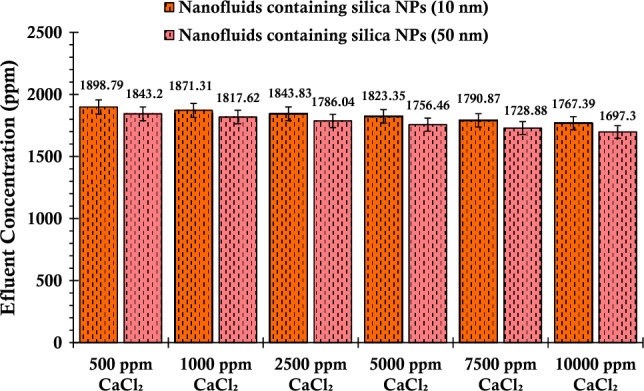
Concentration values of the fluid effluent from the glass bead after the injection of suspensions containing 0.2 wt% of SiO_2_ nanoparticles (with two different nanoparticle sizes) and varying concentrations of CaCl_2_.

As illustrated in Fig. 9, the concentration trends of silica particles within the effluent suspensions extracted from the glass bead, which was impregnated with calcium chloride salt, exhibited a decline corresponding to escalating salinity levels. Notably, as salinity increased from 500 to 10,000 ppm, the particulate concentration within the effluent fluid displayed a notable transition. Specifically, for the suspensions formulated using both smaller and larger nanoparticles, the concentrations shifted from initial values of 1899.79 ppm and 1844.2 ppm to final concentrations of 1766.39 ppm and 1697.3 ppm, respectively. This observed phenomenon underlines the intricate interplay between salinity and particle concentration, shedding light on how the system behaves in response to varying conditions.

**Figure 10:**
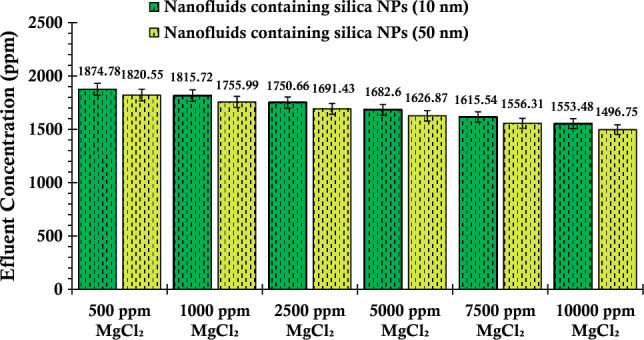
Concentration values of the fluid effluent from the glass bead after the injection of suspensions containing 0.2 wt% of SiO_2_ nanoparticles (with two different nanoparticle sizes) and varying concentrations of MgCl_2_.

In the subsequent investigation, wherein magnesium chloride salt is employed in lieu of calcium chloride salt within the suspensions introduced into the porous media, the outcomes, as depicted in Fig. 10, unveil a notable phenomenon. Particularly, the concentration of silica particles present in the effluent fluid exhibits a remarkable reduction when the salinity level reaches 10,000 ppm of salt. This decrease is particularly stark when compared to the concentration of fluid silica particles at a salinity of 500 ppm salt. For suspensions prepared utilizing both smaller and larger nanoparticles, the observed concentrations stand at 1550.48 ppm and 1494.75 ppm, respectively, under a salinity condition of 10,000 ppm.

**Figure 11:**
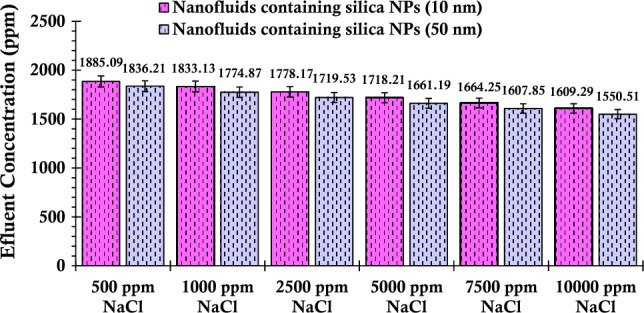
Concentration values of the fluid effluent from the glass bead after the injection of suspensions containing 0.2 wt% of SiO_2_ nanoparticles (with two different nanoparticle sizes) and varying concentrations of NaCl.

Subsequently, sodium chloride salt was introduced into the suspension. As demonstrated in Fig. 11, a similar trend emerges; an increase in salinity leads to a reduction in the concentration of silica particles within the extracted samples. Notably, this pattern holds true for the suspensions crafted from larger silica particles as well. In this scenario, the effluent concentration values of silica particles show a pronounced decrease. This phenomenon serves as an indicator that these larger particles experience a greater degree of entrapment within the porous media, substantiating the complex interaction between particle size, salt type, and porous media behavior.

**Figure 12:**
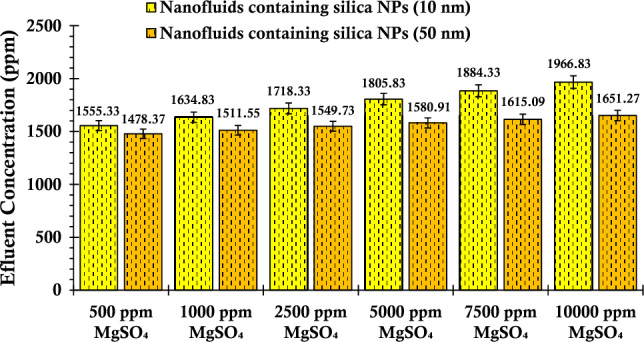
Concentration values of the fluid effluent from the glass bead after the injection of suspensions containing 0.2 wt% of SiO_2_ nanoparticles (with two different nanoparticle sizes) and varying concentrations of MgSO_4_.

Upon scrutinizing the ascending pattern evident in the concentration of silica particles within suspensions incorporating magnesium sulfate salt, as illustrated in Fig. 12, a distinct trend emerges compared to the preceding graphs. With the augmentation of magnesium sulfate salinity, a notable shift in behavior is observed. Specifically, the stability of nanoparticles within the suspension is enhanced, culminating in diminished deposition within the porous media. This outcome consequently contributes to a reduction in particle concentration within the extracted suspensions. The authors' investigation revealed a notable gap in the scientific understanding concerning the interaction of magnesium sulfate salt with silica nanoparticles. To date, no research endeavors have delved into this specific realm with a professional approach. However, drawing parallels from analogous studies, a noteworthy investigation conducted by Li and Cathles explored the influence of sodium sulfate salt—bearing a similar anionic structure to magnesium sulfate salt—on the behavior of silica nanoparticles within suspensions^43^. This particular inquiry highlighted that the inclusion of sodium sulfate salt, examined within a concentration range of up to 100 mM, wielded a substantial impact on both stability enhancement and deposition reduction. These findings allude to the potential efficacy of magnesium sulfate salt, given its similar anionic nature, in mediating comparable outcomes in nanoparticle suspensions.

### Analysis of viscosity values of nanofluids passed through the porous media

**Figure 13:**
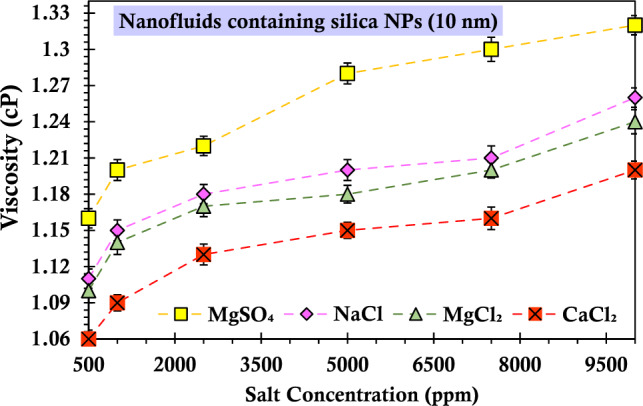
Viscosity values of the suspensions containing 0.2 wt% of SiO_2_ nanoparticles (10 nm) and varying concentrations of salts passing through the glass bead.

**Figure 14:**
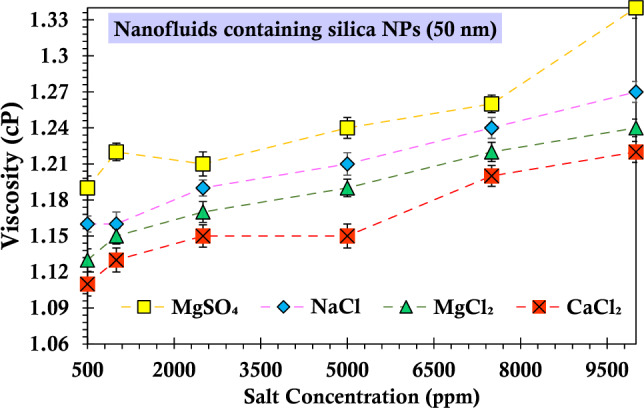
Viscosity values of the suspensions containing 0.2 wt% of SiO_2_ nanoparticles (50 nm) and varying concentrations of salts passing through the glass bead.

Figures 13 and 14 present a comparison between NaCl and other salts containing the same anion (CaCl_2_ and MgCl_2_) in order to measure the influence of ions on the nanofluid's viscosity. The experimental results indicate that, regardless of the silica particle size, the viscosity of the nanofluid is higher for the monovalent cationic ion (sodium) compared to the divalent cationic ion with the same anion. Furthermore, the experimental data presented in the figures demonstrate that the nanofluid's viscosity is greater for the salt containing the sulfate ion compared to the salt containing the chloride ion. Thus, irrespective of the size of silica particles, the nanofluids exhibit an ascending order of viscosity increase as Na > Mg^2+^  > Ca^2+^. The current findings align with those of previous investigators^89^. The ascending trend in nanofluid viscosity can be attributed to the growth in ionic radii of cations in the periodic table group. Elevated charge densities intensify viscosity through hydrogen bond network enhancement and reduced mobilities^90^. Kaminsky elucidated that specific anions (Cl^−^, Br^−^, and I^−^) primarily act as structure-breaking ions, leading to reduced viscosity, while cation hydration (K^+^, Na^+^, and Mg^2+^) increases viscosity^91^. According to Abbas et al., the inclusion of salt in the nanofluid results in a net attractive force, prompting the transition of monomers into dimers, tetramers, and beyond^92^. The measured force at close distances could be attributed to dipole–dipole interactions, leading to the polarization of NPs and electrolyte ions. This increase in structure formation contributes to heightened viscosity. Anions are known to disrupt structures, leading to a decrease in fluid viscosity. However, it has been observed that nanofluids containing ${{\text{SO}}}_{4}^{2-}$ ions exhibit higher viscosity than those with Cl^−^ ions. This is due to a stronger polarization effect of the silanol group caused by the dipole–dipole interaction between ${{\text{SO}}}_{4}^{2-}$ ions and NPs ions compared to the interaction between Cl^−^ ions and NPs ions of the same cation. Consequently, the increase in viscosity of the nanofluid with ${{\text{SO}}}_{4}^{2-}$ ions, as opposed to Cl^−^ ions, can be attributed to the polarization effect induced by the anions.

### Analysis of permeability ratios to gain insights into the underlying mechanisms

In this section, a comprehensive exploration was undertaken to discern the principal and impactful mechanisms governing the injection procedures involving suspensions comprising nanoparticles of varying sizes, formulated under distinct salt concentration conditions. To achieve this, the ratios of permeability diagrams at the conclusive phase to those at the semi-injection phase were meticulously constructed and subjected to in-depth analysis for all suspension variants. This approach offers a systematic insight into the prevailing dynamics, shedding light on the dominant forces dictating the intricate interplay between particle size, salt concentration, and permeability evolution throughout the injection process.

**Figure 15:**
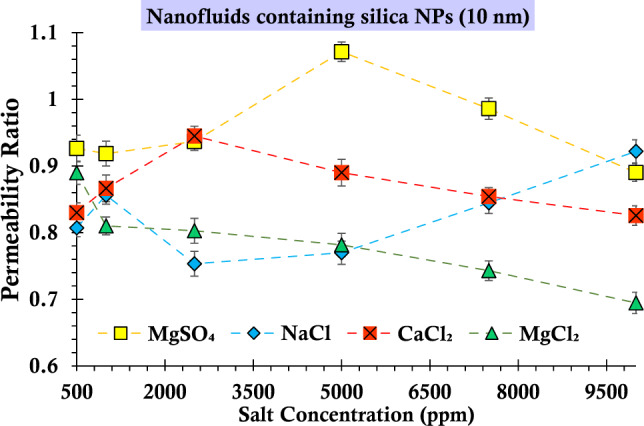
The ratio of final permeability to the middle permeability during the injection process of suspensions containing 0.2 wt% of SiO_2_ nanoparticles (10 nm) and varying concentrations of salts.

As established in previous knowledge, when there is a progressive decline in permeability within porous media over time, anticipation arises that the ratio between the ultimate and interim permeability values will conclusively remain below one. However, Fig. 15 presents an intriguing deviation from this expectation. Notably, for suspensions featuring magnesium sulfate salt at a salinity of 5000 ppm, an unconventional observation was made—the aforementioned permeability ratio exhibited a value exceeding unity, precisely 1.07. This peculiar phenomenon could be attributed to the manifestation of the straining mechanism. Evidently, a sequence of constrictions within the porous structure was occluded by silica particles during the initial phases of injection. This event culminated in a rapid decline in permeability across the injected media. As the injection endured, the operationally of this mechanism subsided, resulting in the reopening of these constrictions. Consequently, the permeability ratio surpassed unity, ultimately reaching the aforementioned value of 1.07. This behavior underscores the intricate interplay between particle interactions and fluid dynamics in porous media, revealing the complex nature of permeability alterations in the presence of specific suspended agents.

**Figure 16:**
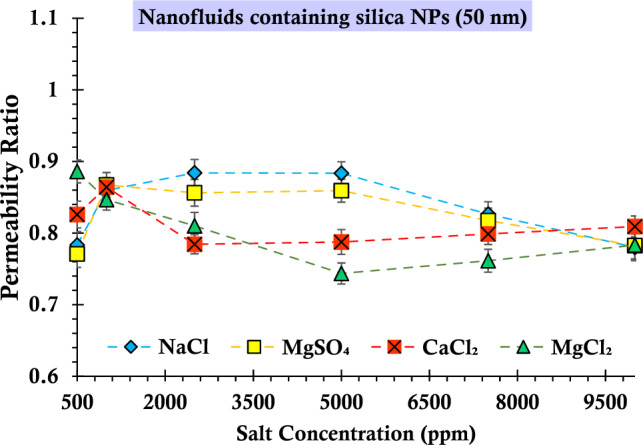
The ratio of final permeability to the middle permeability during the injection process of suspensions containing 0.2 wt% of SiO_2_ nanoparticles (50 nm) and varying concentrations of salts.

An additional noteworthy observation within the graphs depicted in Figs. 15 and 16, pertaining to suspensions formulated using nanoparticles of varying sizes, pertains to the influence of salinity on permeability dynamics. Conventionally, an augmentation in salinity would intuitively align with a diminution in permeability over time, concurrently reducing the permeability ratio. However, upon scrutiny of these figures, a departure from this anticipated pattern becomes evident. Specifically, in the case of suspensions involving smaller silica nanoparticles and featuring magnesium sulfate and sodium chloride salts, as well as for all four salt types in the context of larger silica nanoparticles, the observed decline in permeability is not manifest. This intriguing deviation can be attributed to the prominence of the straining phenomenon^93^. This mechanism operates to an extent where the anticipated correlation between permeability and increasing salinity is disrupted. Consequently, the established notion of permeability ratio consistently decreasing with augmented salinity is challenged due to the overriding influence of the straining mechanism. This complex interplay underscores the multifaceted nature of nanoparticle-salt interactions within porous media, ultimately reshaping our comprehension of the intricate factors governing permeability behavior under varying conditions.

### Examining SEM images to assess the retention of nanoparticles on the glass bead surfaces

**Figure 17:**
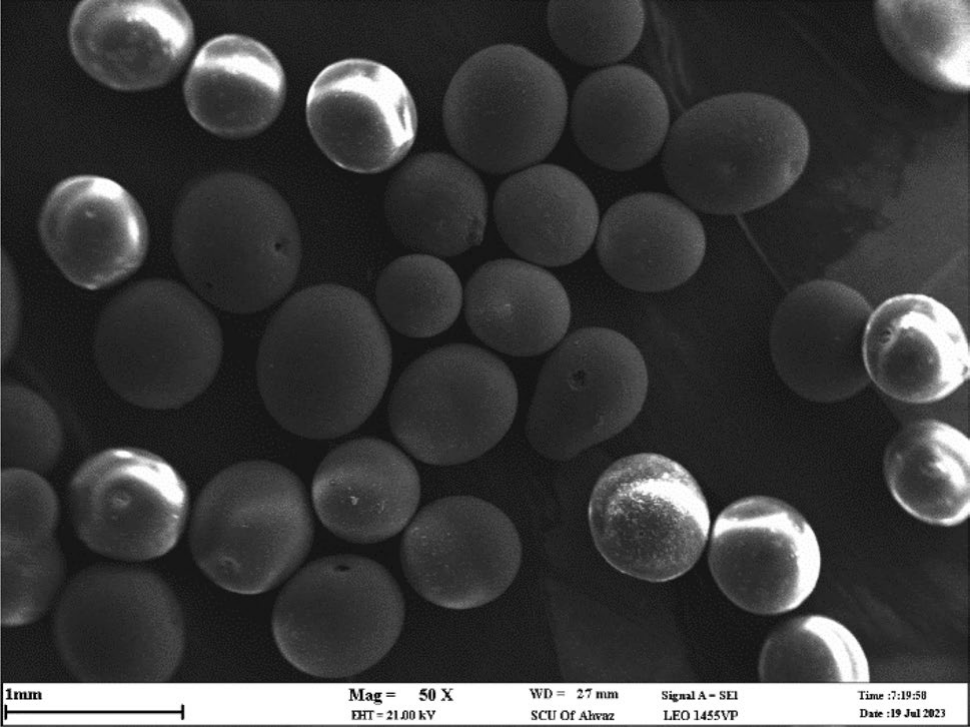
SEM image of clean glass bead used in this research (at ×50  magnification).

This section delves into a comprehensive analysis of scanning electron microscope images portraying glass beads both before and after the introduction of suspensions comprising silica nanoparticles, conducted under varying salt conditions. Illustrated in Fig. 17 is the SEM depiction of the unadulterated glass beads central to this investigation. Notably, the SEM visuals unveil glass beads characterized by nearly spherical geometries.

Subsequently, the focus shifts to the scrutiny of SEM images derived from the surfaces of the glass beads subsequent to the infusion of suspensions. These suspensions encompassed nanoparticles in conjunction with a concentration of 10,000 ppm of salts. Upon the completion of injection and subsequent column extraction, the glass beads were subjected to desiccation. Subsequent to this process, detailed SEM imaging was conducted to document their post-drying morphological characteristics.

**Figure 18:**
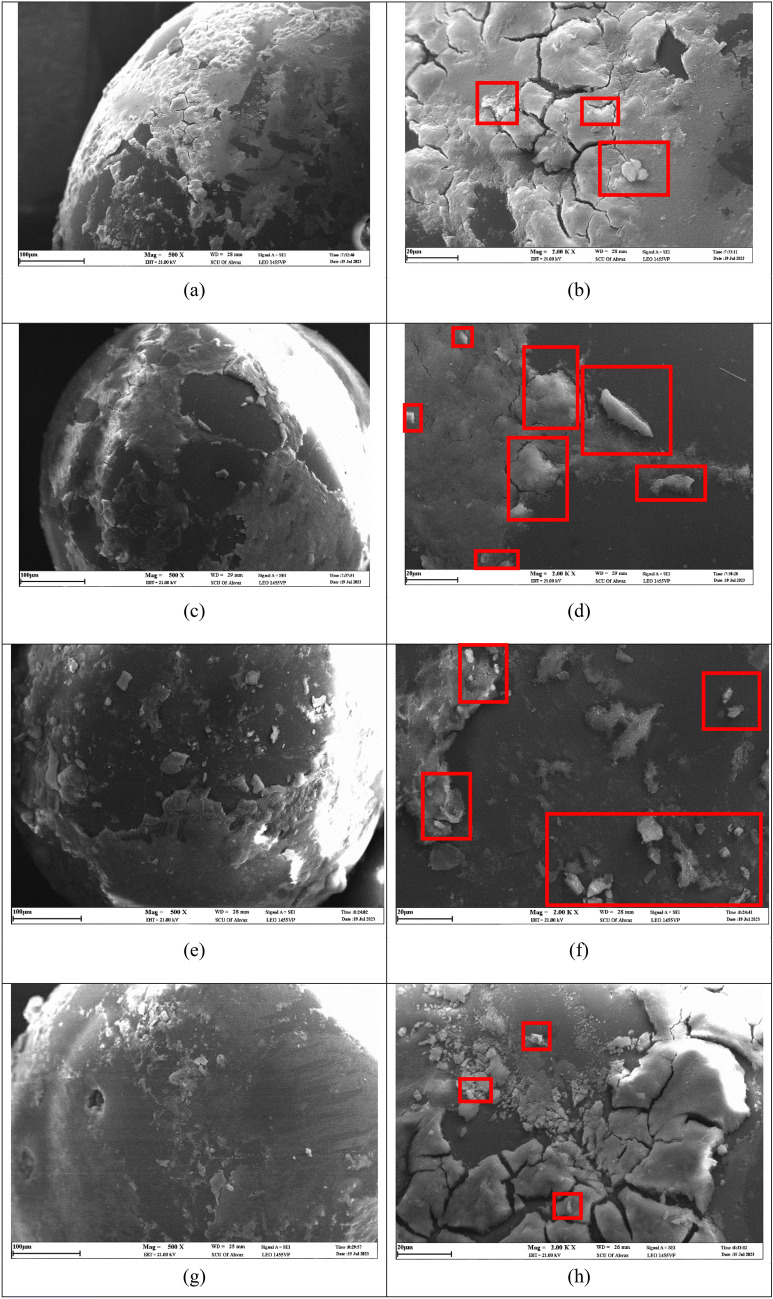
SEM images of the glass bead surfaces after the injection of a suspension containing 0.2 wt% SiO_2_ nanoparticles (10 nm) and 10,000 ppm of CaCl_2_ (**a,b**), MgCl_2_ (**c,d**), NaCl (**e,f**), and MgSO_4_ (**g,h**) at ×500 (left images) and ×2K (right images) magnification.

As depicted in Fig. 18, the SEM images illustrate the existence of desiccated salts and silica particles adhering to the glass bead exteriors. The key observation is that both nanoparticles and salt (in a dried state) are situated on the surfaces of glass beads. When comparing the deposition amount of nanoparticles in the presence of salts on these surfaces, it is essential to draw a clear distinction between them. This distinction is illustrated in Fig. 18, where images taken at 500 (a, c, e, g) and 2000 (b, d, f, h) times magnification reveal the surfaces of the glass beads in the presence of different salts. The areas where nanoparticle accumulations have been deposited are distinctly marked, and in higher magnifications, these areas are highlighted with red boxes. Particularly notable is the augmented deposition of silica particles on the surface when suspensions containing sodium chloride and magnesium chloride salts are introduced. In contrast, suspensions incorporating magnesium sulfate and calcium chloride salts result in a diminished presence of silica particles on the surface. This phenomenon underscores the distinctive interplay between the salt compositions and their influence on the extent of silica particle accumulation on the glass bead surfaces.

**Figure 19:**
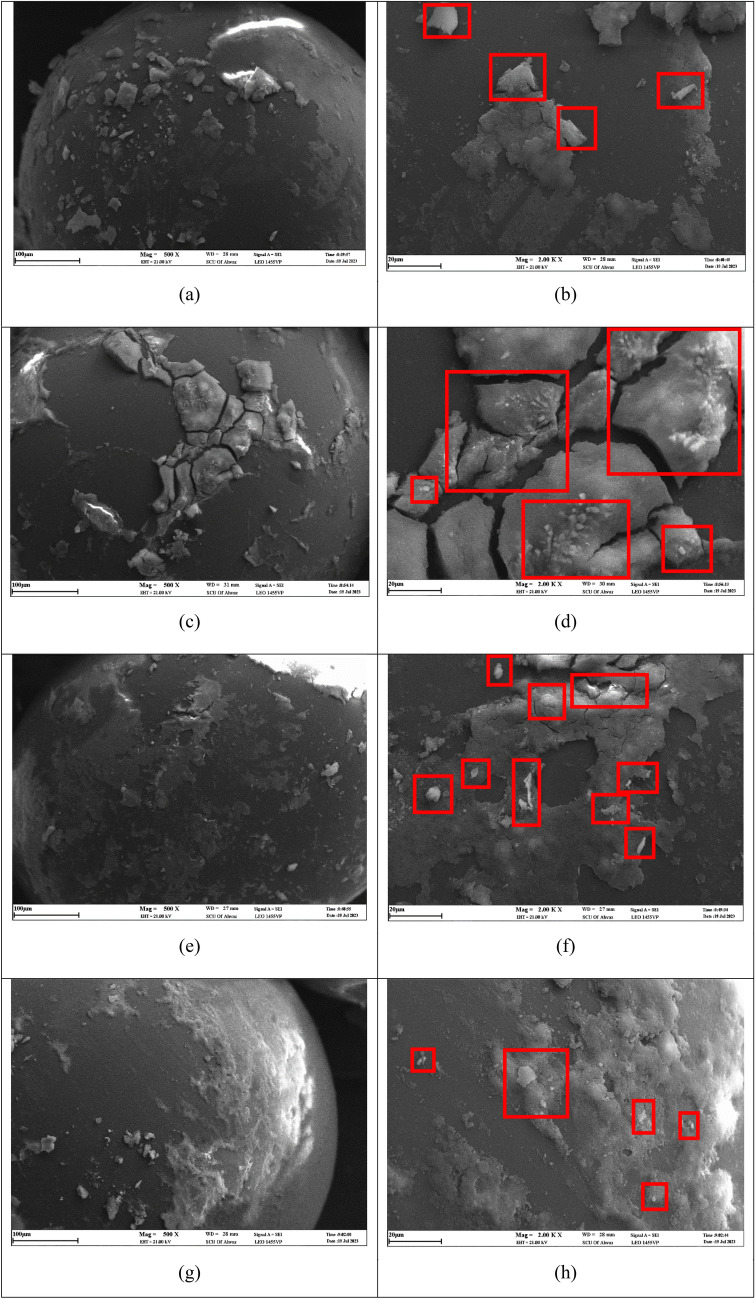
SEM images of the glass bead surfaces after the injection of a suspension containing 0.2 wt% SiO_2_ nanoparticles (50 nm) and 10,000 ppm of CaCl_2_ (**a,b**), MgCl_2_ (**c,d**), NaCl (**e,f**), and MgSO_4_ (**g,h**) at ×500 (left images) and ×2K (right images) magnification.

It becomes evident from Fig. 19 that the utilization of nanoparticles possessing larger dimensions during the suspension formulation process significantly impacts the resultant deposits on the surfaces. The introduction of these larger-sized nanoparticles leads to the creation of increased deposits and surface roughness. Consequently, this phenomenon triggers a notable reduction in permeability, coupled with an increase in the resistance encountered by the flowing media. A salient observation from this analysis is that suspensions containing sodium chloride and magnesium chloride salts tend to exhibit pronounced deposition of silica particles onto the surface under these conditions. This manifestation of enhanced deposition elucidates the intricate interplay between particle size, salt composition, and its consequent influence on surface characteristics and fluid dynamics.

In the final part of this section, it is necessary to mention that this study has offered insights into the complex interplay among silica nanofluids, varying concentrations of salt, and porous media. However, it is essential to acknowledge some limitations in the current study. Our investigation primarily focused on silica nanoparticles, and future studies could explore the behavior of different nanoparticle types to broaden the understanding of diverse systems. Additionally, the experiments were conducted under controlled laboratory conditions, and further research incorporating field-scale parameters would enhance the applicability of the findings.

Despite these limitations, the significance of this work lies in its contribution to the fundamental understanding of nanoparticle transport in porous media. These findings have implications for enhanced oil recovery applications and groundwater remediation processes. Moving forward, future studies could delve deeper into the optimization of nanofluid injection strategies, considering various nanoparticle types and field-scale conditions. This research lays a foundation for advancing subsurface applications, and the engineering insights gained here pave the way for innovative solutions in environmental and energy-related challenges.

## Conclusions

Comprehending the adhesion of nanoparticles to surfaces is crucial for advancing subsurface applications like tracers, groundwater remediation, and enhanced oil recovery. This investigation delves into insights into the stability of dispersion, movement, and retention behaviors of synthesized silica nanoparticles with varying sizes. Conducted within an elongated glass bead column, our study aims to assess the damage caused by nanofluid injection during the formation process. The synthesis process involved generating aqueous silica particle dispersions through the hydrolysis, resulting in silica particles ranging from 10 to 50 nm in size. Employing packed glass bead columns as a surrogate for porous media, our study goes beyond existing research by unraveling the complex mechanisms governing nanoparticle transport and deposition. In contrast to previous works, our comprehensive investigations encompass variations in particle sizes, ionic strength, and ionic species, resulting in the examination of 48 distinct flooding scenarios. Key findings from this research are outlined below.

1. Among the suspensions crafted using various salts, the highest susceptibility to changes in salinity (resulting in a more noticeable decline in nanoparticle aggregation and deposition) was observed in connection with sodium chloride. On the other hand, the least responsive suspension was linked to magnesium sulfate salt, owing to the initiation of the electrostatic stabilization mechanism.
2. The enhanced mechanisms of gravity and log-jamming led to higher retention values in the suspensions having larger nanoparticle dimensions. Notably, the most robust and least significant deposition occurred in nanofluids prepared using magnesium chloride and magnesium sulfate salts, respectively.
3. Owing to the formation of aggregations and subsequent escalated deposition, heightened salinity induces a more pronounced reduction in glass bead permeability. Nevertheless, the prevalence of mechanisms like straining and detachment means that permeability during the injection midpoint might surpass the eventual permeability. This occurrence prompts a transient constriction of throats within the porous media.
4. The outcomes derived from the assessment of porous media permeability subsequent to the introduction of various suspensions was consistent with scanning electron microscope imagery.

## Data Availability

The data will be provided upon request to the corresponding author of this article, R. Daneshfar, via email at reza_daneshfar@ut.ac.ir.

## Abbreviations

SiO_2_: Silica
NP: Nanoparticle
TEOS: Tetraethyl orthosilicate
EOR: Enhanced oil recovery
LSW: Low salinity water
IS: Ionic strength
FEG: Field emission gun
SEM: Scanning electron microscope
FESEM: Field-emission scanning electron microscopy
CaCl_2_: Calcium chloride
MgSO_4_: Magnesium sulfate
MgCl_2_: Magnesium chloride
NaCl: Sodium chloride
XRD: X-ray diffraction
FTIR: Fourier-transform infrared spectroscopy
